# Additional Assessment of Fecal Corticosterone Metabolites Improves Visual Rating in the Evaluation of Stress Responses of Laboratory Rats

**DOI:** 10.3390/ani11030710

**Published:** 2021-03-05

**Authors:** Tina Kroll, Nikola Kornadt-Beck, Angela Oskamp, David Elmenhorst, Chadi Touma, Rupert Palme, Andreas Bauer

**Affiliations:** 1 Institute of Neuroscience and Medicine (INM-2), Forschungszentrum Jülich GmbH, 52425 Jülich, Germany; a.oskamp@fz-juelich.de (A.O.); d.elmenhorst@fz-juelich.de (D.E.); an.bauer@fz-juelich.de (A.B.); 2 Central Animal Facility (ZT), Forschungszentrum Jülich GmbH, 52425 Jülich, Germany; n.kornadt-beck@fz-juelich.de; 3 Department of Psychiatry and Psychotherapy, University Hospital Bonn, 53105 Bonn, Germany; 4 Department of Behavioural Biology, University of Osnabrück, 49076 Osnabrück, Germany; ctouma@uni-osnabrueck.de; 5 Unit of Physiology, Pathophysiology, and Experimental Endocrinology, Department of Biomedical Sciences, University of Veterinary Medicine Vienna, 1210 Vienna, Austria; rupert.palme@vetmeduni.ac.at; 6 Neurological Department, Medical Faculty, Heinrich Heine University Düsseldorf, 40225 Düsseldorf, Germany

**Keywords:** animal welfare assessment, score sheets, fecal glucocorticoid metabolites

## Abstract

**Simple Summary:**

Assessment of animal welfare is an important aspect of preclinical studies to minimize suffering and burden and to improve scientific data. In a standard preclinical setup, such an assessment is normally done via so-called score sheets, which are part of the official documentation and approval of a preclinical study. These score sheets contain different categories, including objective parameters such as animals’ body weight, as well as more subjective criteria such as general status, behavior, and appearance, by which the animal is assessed and given a score reflecting the burden. However, very little is known about whether this mainly visual-based and subjective evaluation of the animals’ welfare reliably reflects the status of the animal and correlates well with more objective parameters used for assessment of animal welfare. To this end, the current study investigates the concordance of parameters obtained via standardized score sheets and fecal corticosterone metabolites in a preclinical neuroscientific setup. Determination of fecal corticosterone metabolites as response parameter of adrenocortical activity is thereby a well-validated parameter often used to determine animals’ stress levels. Our data reveal that specific but subjective scores did not mirror the stress response assessed via fecal corticosterone metabolites in the same animals.

**Abstract:**

Since animal experiments cannot be completely avoided, the pain, suffering, and distress of laboratory animals must be minimized. To this end, a major prerequisite is reliable assessment of pain and distress. Usually, evaluation of animal welfare is done by visual inspection and score sheets. However, relatively little is known about whether standardized, but subjective, score sheets are able to reliably reflect the status of the animals. The current study aimed to compare visual assessment scores and changes in body weight with concentrations of fecal corticosterone metabolites (FCMs) in a neuroscientific experimental setup. Additionally, effects of refinement procedures were investigated. Eight male adult Sprague-Dawley rats underwent several experimental interventions, including electroencephalograph electrode implantation and subsequent recording, positron emission tomography (PET), and sleep deprivation (SD) by motorized activity wheels. Additional 16 rats were either used as controls without any treatment or to evaluate refinement strategies. Stress responses were determined on a daily basis by means of measuring FCMs, body weight, and evaluation of the animals’ welfare by standardized score sheets. Surgery provoked a significant elevation of FCM levels for up to five days. Increases in FCMs due to PET procedures or SD in activity wheels were also highly significant, while visual assessment scores did not indicate elevated stress levels and body weights remained constant. Visual assessment scores correlate with neither changes in body weight nor increases in FCM levels. Habituation procedures to activity wheels used for SD had no impact on corticosterone release. Our results revealed that actual score sheets for visual assessment of animal welfare did not mirror physiological stress responses assessed by FCM measurements. Moreover, small changes in body weight did not correlate with FCM concentration either. In conclusion, as visual assessment is a method allowing immediate interventions on suffering animals to alleviate burden, timely stress assessment in experimental rodents via score sheets should be ideally complemented by validated objective measures (e.g., fecal FCM measured by well-established assays for reliable detection of FCMs). This will complete a comprehensive appraisal of the animals’ welfare status in a retrospective manner and refine stressor procedures in the long run.

## 1. Introduction

To date, several methodological alternatives, such as cell cultures, computer simulations, and biochips, have been investigated as potential replacements of animal experiments. However, if an intact organism is necessary to fully elucidate the functions and interactions of specific systems—e.g., in neuroscience—animal experiments cannot be completely avoided. One of the major principles for animal use in scientific research is the principle of replacement, reduction, and refinement (3Rs). This principle was first described by Russel and Burch in 1959 with the aim of ensuring the least harmful treatment as possible for laboratory animals to conduct successful experiments [1]. Many years later, the 3Rs were implemented within the European Law by the EU Directive 2010/63 dealing with the protection of animals used for research [2]. As animals in research could not be completely replaced, the principle of refinement should minimize the pain, suffering, and distress of laboratory animals. However, a major prerequisite is a reliable method to recognize pain and distress in animals since they cannot report verbally as humans do. Accordingly, investigators, animal care staff, and veterinarians are in charge of a reliable and well-performed observation of experimental animals [3]. A first attempt to compile observable criteria to describe the abnormal behavior of laboratory animals indicating suffering and distress was made by Morton and Griffith [4]. Since then, a great deal of effort has been put into the development of a reliable and objective severity assessment, including the release of a guideline to facilitate the implementation of the Directive 2010/63/EU with regard to severity assessment by an expert working group established by the European Commission [5]. Accordingly, national legal authorities are progressively demanding visual assessments of laboratory animals on the basis of categorized score sheets, which are regularly part of the permission for conducting animal experiments. However, assessment of animals’ welfare is often not the main focus of the experiment and it can be questioned whether individual score sheets and observations are able to reliably reflect the status of the animals. This is of special interest with regard to the assessment of animal welfare, definition of adequate endpoints, retrospective severity reporting increasingly demanded by the local authorities, and scientific outcomes, as well as publication [3]. In addition, stress causes multiple behavioral and physiological alterations which might influence scientific results [6]. Thus, a careful collection of observational data is necessary for analyzing in vivo animal research data in a broader context and for contributing to accuracy and the generation of novel hypotheses [3]. Observation and reporting of stress responses in laboratory animals due to distinct experimental interventions are gaining more and more importance [7,8,9,10,11,12,13,14,15] but, to date, multidimensional approaches to severity assessment are rare and evidence-based severity assessment is still challenging (for review, see Keubler et al. [16]). Most studies have relatively short observation times and assessment of one single experimental intervention in common. Moreover, assessment of distress is often performed via different parameters (behavior, body weight, fecal corticosterone metabolites (FCMs)) but investigations either did not focus on concordance of the parameters (e.g., [7,8,9,17,18]) at all or only did so in a limited manner [19,20,21]. Only recently, some research groups have worked on a multidimensional evaluation of burden in animal experiments, e.g., via cluster analysis of body weight and wheel running behavior, but individual and subjective scoring parameters were not the focus of the experiments and, most often, mice were used [22,23]. In the current study, rats longitudinally underwent several experimental conditions in the context of a preclinical study. Interventions included surgery (electroencephalograph (EEG) electrode implantation) under isoflurane anesthesia followed by a one-week post-operative monitoring, subsequent EEG-recording in dedicated cages, positron emission tomography (PET) scans under isoflurane anesthesia, and sleep deprivation (SD) in activity wheels. During experimental conditions, stress responses and animal welfare were evaluated by visual assessment scores via categorized score sheets and determination of body weight. Additionally, feces were sampled for measuring corticosterone metabolites as this is an essential component of the stress response in mammals via activation of the hypothalamic–pituitary–adrenal (HPA) axis. Final products of the HPA axis are glucocorticoids (GC) synthetized in the adrenal glands (for review, see Nicolaides et al. [24]). Corticosterone is the ultimate GC in the rat, metabolized in the liver and excreted via urine and feces (for review, see Möstl and Palme [25]). During recent years, quantification of corticosterone metabolites in feces has become an increasingly important, non-invasive tool offering the advantage of long-term monitoring of stress responses in laboratory animals [8,11,12,26,27]. In the current study, a group-specific and well-validated enzyme immunoassay (EIA, [28,29,30]) was used to monitor the stress responses of the animals during experimental interventions.

By comparing different outcome parameters—determined in the same animals at the same timepoints—the present study focuses on the concordance and reliability of assessment of animal welfare during experimental interventions in a preclinical neuroscientific setup. In particular, the assessment by given scores based on subjective ratings, done by varying personnel in a daily routine, using categorized score sheets is reviewed. In addition, effects of refinement procedures in the form of specialized habituation to the used activity wheels (for inducing sleep deprivation) were investigated.

## 2. Materials and Methods

### 2.1. Animals and Study Design

Adult male Sprague-Dawley rats (Charles River Laboratories, Sulzfeld, Germany), *n* = 24, mean ± standard deviation; body weight: 523 g ± 41 g; age: >3 month) were used. All experiments were performed in spring in two subsequent cycles with 12 animals each. All rats within one cycle were housed in the same room during the study, under stable environmental conditions (temperature 21 °C ± 1 °C) and with access to food (ssniff Spezialdiäten GmbH, Soest, Germany) and demineralized water ad libitum. Macrolon cages were cleaned once a week and wooden chips (aspen wood, AsBe-wood GmbH, Hamburg, Germany) were used as bedding material. Animals were provided with nestlets (cotton squares, 5 × 5 cm, ssniff Spezialdiäten GmbH) and wooden pieces for gnawing (aspen wood, 5 × 2 × 2 cm, Plexx B.V, Elst, The Netherlands). Other types of enrichment, such as tunnels, could not be used because standardized experimental sleep deprivation is essentially dependent on a strictly controlled dark–light cycle. All materials were autoclaved before usage. All efforts were made to disturb the animals as little as possible and to minimize the number of personnel handling the animals.

All experiments were conducted in accordance with the German Animal Protection Act and approved by regional governmental authorities (Landesamt für Natur, Umwelt und Verbraucherschutz Nordrhein-Westfalen, AZ84-02.04.2011.A136).

Directly after delivery, rats were housed three per cage (floor space 1800 cm^2^) and adapted to a 12-h light–dark cycle with lights on at midnight. Shifting to this new light–dark cycle was performed over three days, starting with lights-on after delivery in order to give the animals the opportunity to sleep after transportation in darkness. During the shift to the new light–dark cycle (midnight to noon), the lights-off periods varied between 10.5 and 12 h whereas lights-on periods accounted for 10 to 13 h.

On day 4, rats were randomly assigned to three experimental groups consisting of eight animals each (four per cycle): group 1 (G1, test group): 8 rats undergoing EEG-electrode implantation and subsequent EEG-recording in dedicated cages followed by a PET measurement and 24-h SD with EEG-recording in motorized activity wheels; group 2 (G2, control group): 8 rats housed in EEG-cages without recording and subsequent 24-h pseudo-SD in motorized activity wheels after extensive habituation to the wheels; group 3 (G3, activity wheel control group): 8 rats subjected to 24-h pseudo-SD in motorized activity wheels after a short habituation to the wheels (1 h on two consecutive days). All animals were single-housed in macrolon rat cages with a floor space of 810 cm^2^ (if not otherwise indicated) during interventions.

**Figure 1:**
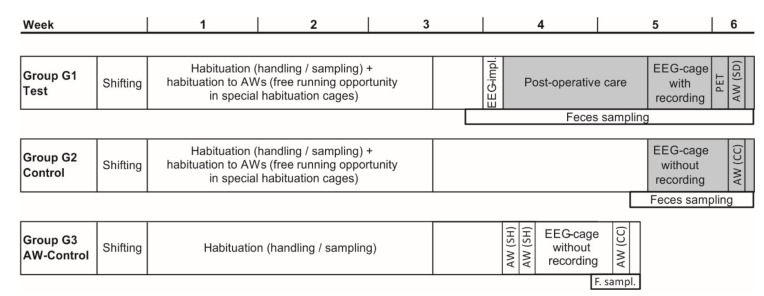
Experimental design. Grey-colored areas indicate time periods of visual scoring. Each group *n* = 8. AW, activity wheel; CC, control condition; EEG, electroencephalogram; PET, positron emission tomography; shifting, adaptation to the light cycle with lights on at midnight; SD, sleep deprivation; SH, short habituation (1 h on two consecutive days).

For further details on the experimental design, see Figure 1.

### 2.2. Habituation

**Figure 2:**
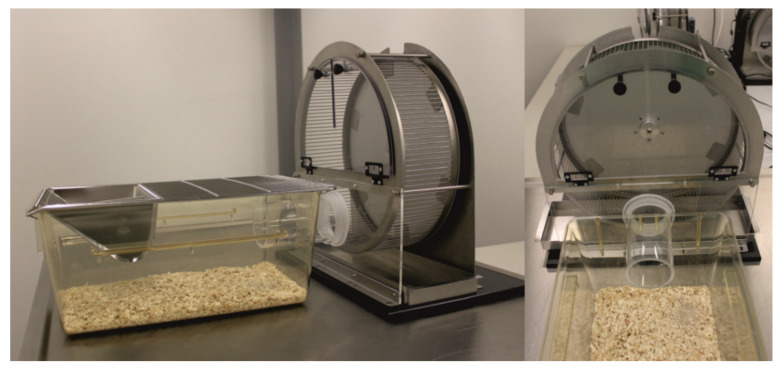
Cages (810 cm^2^) connected to activity wheels for free-running opportunities during habituation. In the course of experiments, cages were removed and wheels were used for (pseudo-) sleep deprivation.

Rats in G1 and G2 (*n* = 8 each) were single-housed in cages connected to activity wheels (see below) by a short acrylic glass tunnel (see Figure 2) for 17 days. Wheels were unlocked so that rats had the opportunity of free-running for habituation purposes.

Rats in G3 (*n* = 8) were initially group-housed in standard rat cages (floor space 1800 cm^2^) and separated 9 days before the start of the experiments in order to have the same housing conditions in all groups during experimental interventions but minimize the burden that the animals are exposed to. Thereby, 5 days after separation, these rats were subjected to a short 1-h wheel habituation (start at 11 AM) with a fixed running schedule (5 min off/5 min on at a speed of 3 m/min, [31]) on two consecutive days.

During the first three weeks of the experiment, all rats were accustomed to feces collection and handling, including gentle fixation and weighing (5 min each day during the first week), in order to minimize animals’ stress during the following experimental procedures (for details of the sampling procedure, see below).

### 2.3. EEG-Electrode Implantation and Recording

Animals in G1 were implanted with EEG-electrodes (Pinnacle Technology INC, Lawrence, KS, United States) at the beginning of week 4, according to the manufacturer’s protocol, under aseptic conditions. Anesthesia was induced with 5% isoflurane in 2 L O_2_/min in an induction chamber. For analgesia, animals were provided with 5 mg/kg body weight carprofen (Rebopharm, Bocholt, Germany) subcutaneously (s.c.). Painful interventions (e.g., trepanation) were conducted at the earliest 30 min after medication and under additional local anesthesia (Lidocard HCl 2%, B. Braun Melsungen AG, Melsungen, Germany)). During the whole surgery, physiological parameters were carefully monitored (Pulse Oximeter Sense, Uno Roestvaststaal BV, Zevenaar, The Netherlands). Depth of anesthesia was controlled and adapted via breathing rate (frequency between 40 and 50/min, 2–2.5% isoflurane in 2 L O_2_/min). After preparation of the skull, four small drill holes were placed at specific coordinates (frontal cortex: +2 mm antero-posterior (AP), +2 mm medio-lateral (ML), parietal cortex: −4 mm AP, −1 mm ML and cerebellum: −6.5 mm AP, +/−3 mm ML), recording screws were inserted and soldered to the headmount. Dental acrylic (GC FujiCEM 2, Dentabo OHG, Böhringen, Germany) was used for fixation of screws and headmounts, and wounds were covered with synthetic skin replacement (Epigard^®^, Medisafe GmbH, Hamburg, Germany) for better wound healing. Total anesthesia time during EEG-surgery was approximately 1.5 h.

For analgesia, on post-operative days 1 and 2, 5 mg/kg body weight carprofen was given s.c. Food and water intake was supported by a glucose gel-pad (Solid Drink^®^, HG, Tiel, The Netherlands) for two days after surgery.

**Figure 3:**
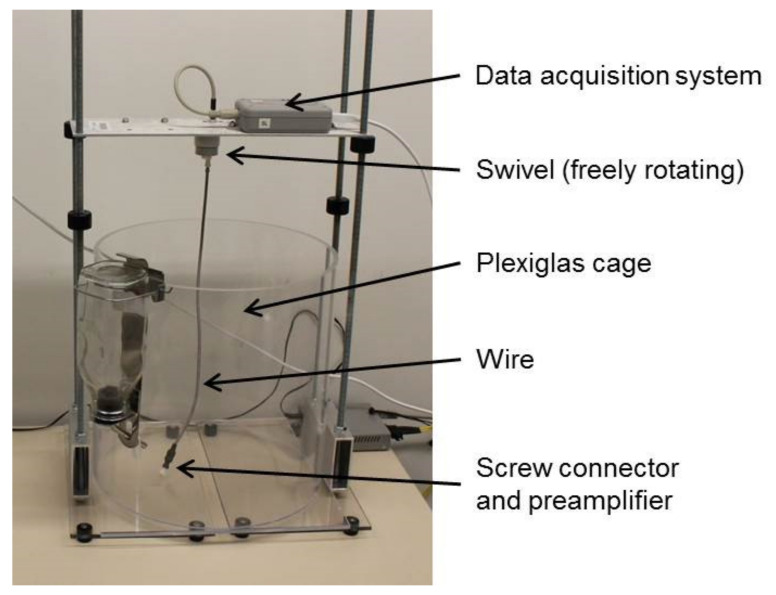
Acrylic glass EEG-cage used for EEG-recording.

Eight days after surgery, animals were connected to the tethered EEG-recording system (between 11 AM and noon) and data were recorded until the end of the experiments. During recording, before and after the SD procedure (see below), animals were housed in round (diameter 35.5 cm) acrylic glass EEG-cages (8273 rat cage, Pinnacle Technology INC, Lawrence, KS, USA). Cages were covered one half each with a towel to darken the cage. A swivel allowed for free movement (see Figure 3). Control rats (G2 and G3) were housed in the same EEG-cages but without any cable connection and data recording.

### 2.4. Positron Emission Tomography

For transportation to the imaging facility, animals were placed in their home cages in tight styrofoam boxes. Upon arrival, anesthesia was induced with 5% isoflurane in 2 L O_2_/min and maintained with 2% isoflurane in 2 L O_2_/min. A tail vein catheter served for radiotracer injection followed by a 70-min PET measurement (Siemens Inveon Multimodality PET scanner (Siemens, Knoxville, TN, USA) as previously described in [32]). Total anesthesia time was around 2.3 h. After being wide-awake, animals were transported back to the animal facility and were reconnected to the EEG-recording system.

### 2.5. Sleep Deprivation (SD)

SD in rats of G1 was performed in motorized, stainless-steel wheels (rat tethered motorized wheel model 80860A, Lafayette Instrument, Lafayette, IN, USA) under continuous EEG-recording. The 24-h SD procedure started at noon with the first 12 h in the animals’ active phase (lights off) and was ensured by forced running (3 s “on” at a speed of 3 m/min and 12 s “off”; activity wheel control and counter model 86070A, Lafayette Instrument, resulting in 288 min activity in 24 h. Similar parameters have been previously shown to produce greater than 93% wakefulness [33,34]. Rats in control groups G2 and G3 underwent motor control condition (pseudo-SD) with a running schedule of 36 min constant running at a speed of 3 m/min in each 3-h period, resulting in the same locomotor activity over 24 h but with the opportunity of deep sleep [31].

### 2.6. Welfare Assessment and Body Weight

**Figure 4:**
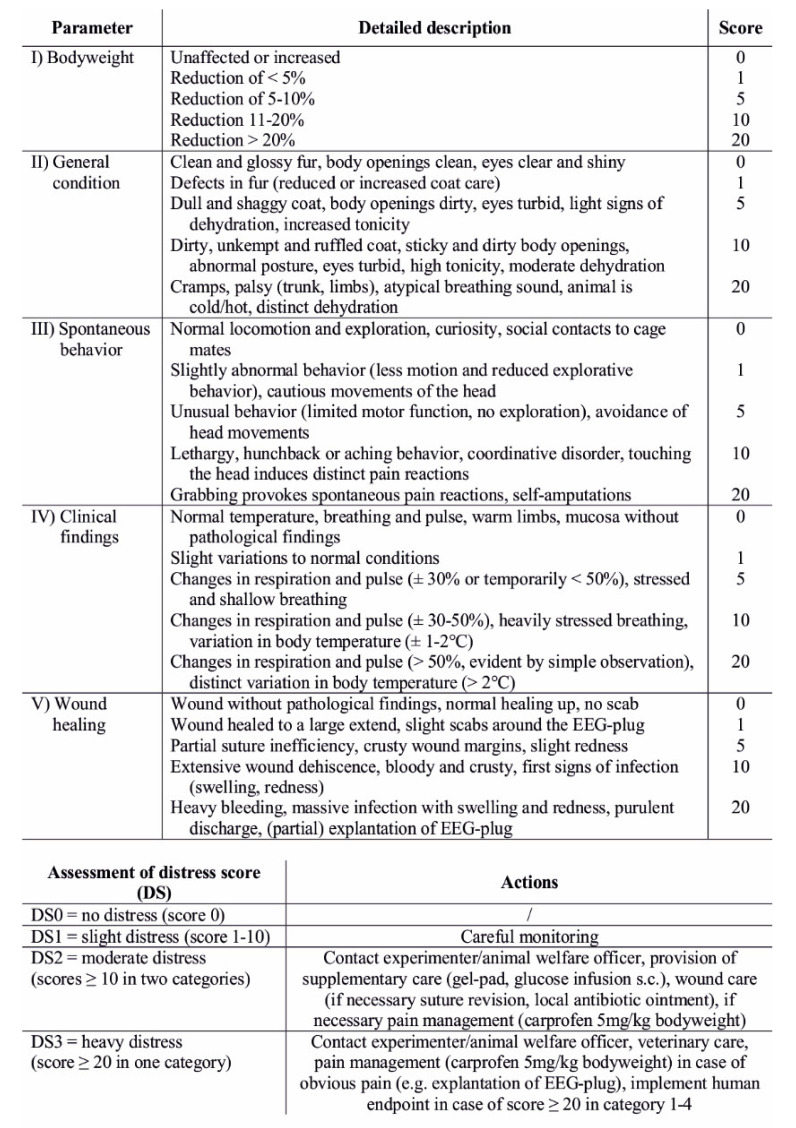
Score sheet used for assessment of the animals’ welfare (modified from [35,36]) including the categories body weight, general condition, spontaneous behavior, clinical findings, and wound healing.

Rats’ welfare during the post-operative period (after surgery, for one week) and sleep deprivation was assessed daily and at the same time (between 11 and 12 AM) on the basis of standardized score sheets, including the categories of body weight, general state of health, spontaneous behavior, clinical findings, and wound healing (see Figure 4). Standardized scores (0, 1, 5, 10, and 20 [35,36]) were given for all these categories, leading to a summarized general stress score with a maximum of 100. During scoring, animals were first observed in their home cages in order to detect abnormalities in general appearance (coat and grooming, eyes, skin, breathing), spontaneous activity including posture, and social behavior, which was mainly evaluated by attempts to make contact with mates in cages placed next door. Location of cages did not change during the time course of experiments and thus mates in adjacent cages remained the same. Next, animals were taken out of their home cages for weighing. During handling, responses to manipulation (vocalization, lethargy) and clinical signs such as altered body temperature, changes in heart frequency as well as hydration status were evaluated qualitatively by careful observation. Explicit measurement of body temperature was only performed if the body temperature of an animal appeared to be much colder or warmer than normal during handling. Last, surgical wounds were assessed with regard to healing, crusting, swelling, redness, bleeding, dehiscence, and explantation of the EEG-recording system. During EEG-cage housing, animals were scored accordingly but were not taken out of the EEG-cages in order to avoid additional stress and bias in EEG-data due to unplugging and reconnecting to the EEG-recording system. During EEG-cage housing, animals’ weights were only assessed before start (EEG1) and after end of recording (EEG5).

Categorization of general summarized scores was as follows: 0 = no stress, 1–10: light stress, >10 in two of the assessed categories/parameters: moderate stress, >20 in one category: severe stress. In case of moderate stress scoring, contact with the experimenter and/or the animal welfare officer was mandatory as well as supportive treatment with additional gel-pads, glucose infusions (s.c.), pain medication (carprofen 5 mg/kg once per day), and/or wound treatment depending on the observed abnormalities. Severe stress resulted in pain medication in the case of obvious pain (e.g., explantation of the EEG-recording system) and/or veterinary advice and treatment. Loss of body weight >20% in relation to preoperative weight, cramps, paralysis, self-amputations, distinct changes in respiration, pulse, and body temperature (see Figure 4) as well as uncontrollable bleeding and infections directly led to euthanasia.

Ratings were made by one single person per day (physician, technician, keeper, or veterinarian). All staff were well-trained in the assessment of (ab)normal behavior in laboratory rats and were instructed regarding how to score the animals under investigation in advance. Observers did not undergo an interrater reliability assessment as it was the intention to mirror the daily routine of a preclinical laboratory and animal facility at which usually different, but trained, raters evaluate animals’ welfare according to standardized schemes without a priori assessment of interrater reliability.

### 2.7. Fecal Corticosterone Metabolites (FCMs)

First, 24 h before starting feces sampling, all “old” feces were manually removed from the cages with forceps. Cages were not changed in order to prevent stress due to new housing conditions. During experiments, all fecal pellets voided were collected with forceps once a day between 11 and 12 AM and in addition between 11 and 12 PM after (pseudo-) SD. Sampling started one day before surgery (G1) and one day before housing in EEG-cages (G2) or activity wheels (G3), respectively. FCM concentrations from samples collected at days 1 and 2 of the experiments were averaged and served as individual baseline (BL) of FCM levels. Altogether, 291 fecal samples were collected and immediately stored at −20 °C until further processing.

Fecal samples were thawed and placed in a drying oven (80 °C) for 3–5 h. Dried feces were crushed and carefully mixed. A portion of 50 mg of each crushed feces was mixed with 1 mL of 80% methanol, followed by vortexing (30 min) and immediate centrifugation (2500 g, 15 min) [29,37]. A 500-µL aliquot of the resulting supernatant was frozen (−20 °C) until analysis with a 5α-pregnane-3β,11β,21-triol-20-one enzyme immunoassay [29]. This EIA has been successfully validated for monitoring adrenocortical activity in laboratory rats and mice [28,30,38].

### 2.8. Statistical Analysis

All values are reported as mean ± standard deviation. FCM values are given in ng/0.05 g feces. Body weight is given in g. Percentage relative differences in body weight and FCM levels are related to individual baseline values (normalized to 100%) unless otherwise noted.

Differences in baseline FCM levels and body weight between groups were evaluated with an analysis of variance (ANOVA). Time-courses of FCMs and body weight were investigated with a mixed-model analysis of variance (rmANOVA) with FCMs during each period or body weight treated as within-subject factor and, if appropriate, different groups of animals as between-subject factor. *p* < 0.05 was regarded as significant and data were further evaluated with post-hoc *t*-tests (two-tailed or paired dependent on the variables) as well as subsequent Bonferroni correction. Pearson’s product moment correlation was used to examine all correlations, except the relationship between individual FCM values and overall visual assessment scores, for which Kendall’s tau rank correlation coefficient was determined.

All statistical analyses were conducted with SPSS Software v.22 (SPSS Inc., Chicago, IL, USA).

SD in rats of G1 took place in the second half (12–24 h) of the 24-h time period in the activity wheels. The corresponding 12-h sampling period with sampling time at midnight solely represents the inactive, sleep-deprived phase of the animals due to the delay in FCM excretion [28] and is likely biased due to a circadian rhythm of corticosterone secretion [26] in comparison to the standard 24-h sampling period. Previous experiments on the circadian rhythm of FCMs in the same animals showed that FCMs sampled at midnight were on average 18% higher in comparison to FCMs displaying the mean of the active and inactive phase (sampling period 24 h) of the animals (for further information please see File S1, Table S1, and Figure S1). In order to make values comparable to baseline, FCM values depicting solely the inactive phase of the animals (SD in G1, sampled at midnight) were adjusted by −18%. Whenever direct comparisons between G1 and G2 were made, the corresponding pseudo-SD FCM values in G2 were equally adapted.

## 3. Results

### 3.1. Baseline (BL) Values

To determine the robustness of baseline values, FCM concentrations on both experimental days were analyzed with regard to outliers. Analysis revealed BL FCM levels of one rat in the test group on day 1 as an outlier (more than three times higher compared to the mean of all rats of the same group). As the BL FCM level of this rat on day 2 was still twice as high as the mean of the group, data of this rat were excluded from all further analyses.

Comparison of BL values on day 1 and day 2 per group (paired *t*-test, *n* = 7/8) did not reveal significant differences between the two experimental days. BL FCM concentrations of all groups did not differ significantly and ranged from 966 ± 316 (test group) to 1103 ± 314 ng/0.05 g (activity wheel control group). Mean body weights of the animals per group directly before interventions were 527 ± 42 g in the test group, 563 ± 43 g in the control group, and 483 ± 23 g in the activity wheel control group, respectively. Comparison of body weights between groups indicated a significant variation (*p* = 0.001). Post-hoc analysis revealed a significant difference between the two control groups with G2 > G3 (*p* < 0.001).

### 3.2. Concentration of Fecal Corticosterone Metabolites during Experimental Interventions

**Figure 5:**
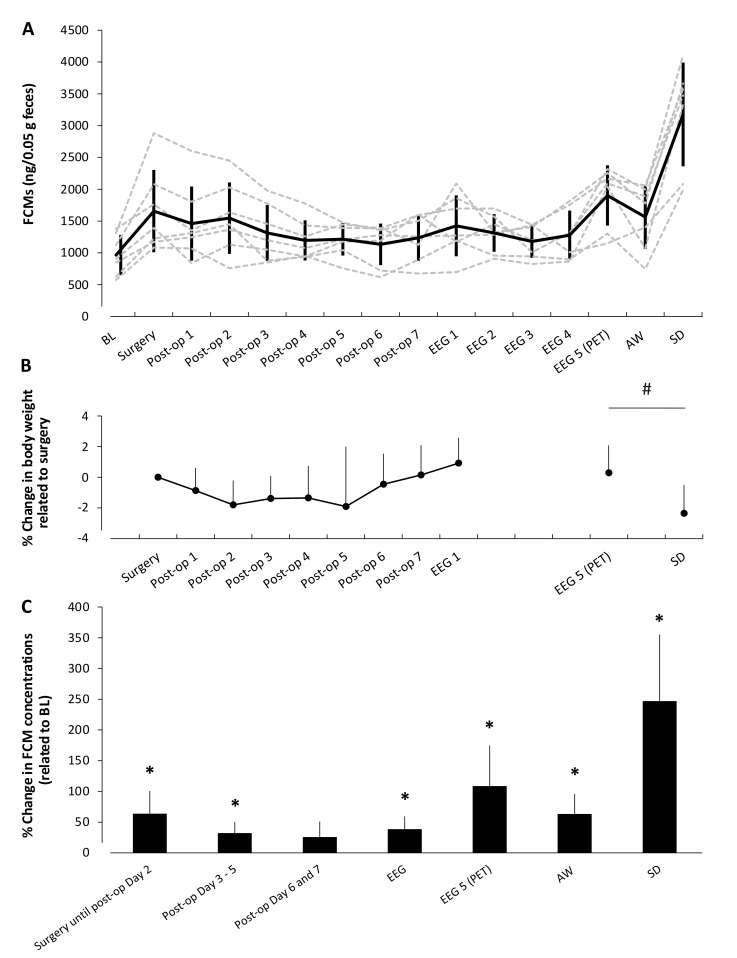
Concentrations of fecal corticosterone metabolites (FCMs) and development of body weight over the course of experiments in the test group G1. Interventions comprised electroencephalogram (EEG) electrode implantation, EEG-recording, imaging with positron emission tomography (PET), and housing in rotating activity wheels (AW) during the active (lights-off) and inactive (lights-on, corresponding to SD) phase of the animals (*n* = 7). (**A**) Dotted lines depict individual time courses of FCMs; the solid line represents the mean FCM concentration ± standard deviation. (**B**) Development of weight over time given as relative change in relation to body weight at the day of surgery. # indicates significant differences in body weight between pre- and post-SD condition. (**C**) Percentage difference of FCMs during interventions related to respective baseline (BL) values. * indicates significant differences in comparison to baseline values set to 100% by post-hoc comparisons (paired *t*-tests on a Bonferroni corrected level of significance of *p* < 0.007). Error bars denote standard deviations.

Figure 5 shows the time courses of individual FCMs (Figure 5A), the development of body weights over time (Figure 5B), and the mean relative differences per period in FCMs related to BL (Figure 5C) in seven rats undergoing the described experimental conditions.

Overall mixed-model analysis of variance indicated a significant influence of period/experimental procedure (F (1.5, 9) = 21.7, *p* = 0.001). Bonferroni-corrected paired *t*-test (adapted level of significance *p* < 0.007) revealed significantly increased FCM levels in response to EEG-electrode implantation in all rats during the early post-operative period, including supply with analgesics on days 1 and 2 (63.8 ± 37%, post-hoc test: *p* = 0.0039). Initial FCM responses were highly individual but, in nearly all animals, absolute FCM concentrations decreased after the second post-operative day (end of analgesia via s.c. injections) and interindividual differences between animals became smaller (average % coefficient of variance (COV) surgery until post-op day 2: 37.4; average %COV post-op days 3—5: 26.2%). Nevertheless, FCM levels remained significantly elevated in comparison to the pre-operative status in the middle post-operative period (days 3—5: 32.1 ± 18%, post-hoc test: *p* = 0.0033). During the late post-operative period, from day 6 onwards, FCM levels stabilized (post-hoc test: *p* = 0.024) to slightly but not significantly elevated levels (days 6 and 7: 25.8 ± 25.2%).

After connection to the EEG-recording system, FCM concentrations again rose by 13.4 ± 38.1% in comparison to post-op days 6/7 (paired *t*-test *p* = 0.4). During the days of EEG-recording, FCM levels normalized towards middle post-operative values but were still significantly higher in comparison to BL FCM concentrations (EEG days 1–4: 38.7 ± 20.7%, *p* = 0.0026). A further considerable but, again, per animal individually variable increase in FCM levels (108.6 ± 66.2% related to BL, *p* = 0.005) was observed on the day of PET imaging.

Subsequent housing in rotating activity wheels during the active, lights-off phase of the animals (forced running) led to significantly elevated FCM concentrations (63.4 ± 32.5%, *p* = 0.002) in the range found at early post-operative days. Most strikingly, the magnitude of the FCM increases due to SD in scheduled activity wheels was highly significant (247 ± 108.3% related to BL, *p* = 0.0009) and several times higher than the FCM increase in response to surgery.

### 3.3. Concentration of Fecal Corticosterone Metabolites in Relation to Different Housing Conditions

#### 3.3.1. EEG-Cage Housing (Control Group)

**Figure 6:**
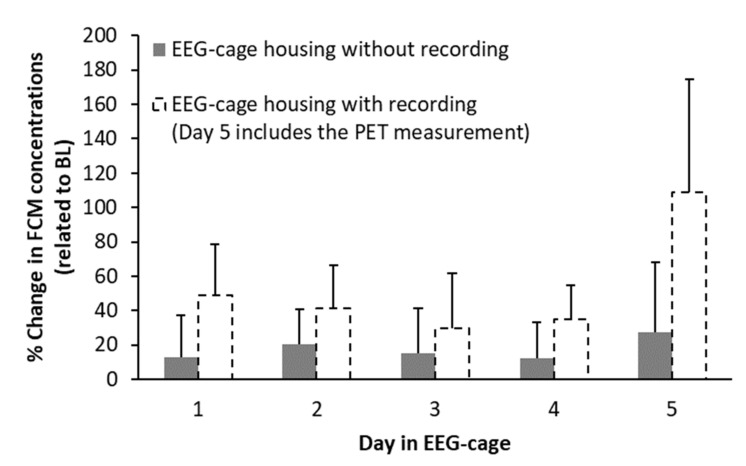
Difference (% change) in fecal corticosterone metabolite (FCM) concentrations during electro-encephalographic (EEG-) cage housing without EEG-recording related to baseline (BL) values (control group, *n* = 8). For comparison, dotted columns depict FCM levels of rats with EEG-recording and a positron emission tomography (PET) measurement at Day 5 (test group, *n* = 7). Error bars denote standard deviation.

Figure 6 demonstrates the increase in FCM levels during EEG-cage housing without EEG-recording (normalized to BL values set to 100%). Changes in FCM levels due to housing in the specialized EEG-cages were subtle and not significant (range from 12.5 ± 24.5% (Day 1) to 27.2 ± 41% (Day 5)). As FCM concentrations in animals with EEG-electrode implantation and subsequent recording (test group) were not significantly elevated during late post-operative care (directly before EEG-cage housing and EEG-recording; see above), an explorative comparison between groups was performed. Overall, repeated-measures ANOVA indicated significant differences between groups (F(1, 13) = 9.8, *p* = 0.008). However, these differences did not withstand post-hoc comparisons with a Bonferroni-corrected level of significance of 0.01, but showed a strong trend towards significance on the first (*p* = 0.02, connection to EEG-recording system in the test group) and the last day (*p* = 0.012, PET measurement in the test group) of the experiment.

#### 3.3.2. Housing in Activity Wheels and Effects of Habituation

**Figure 7:**
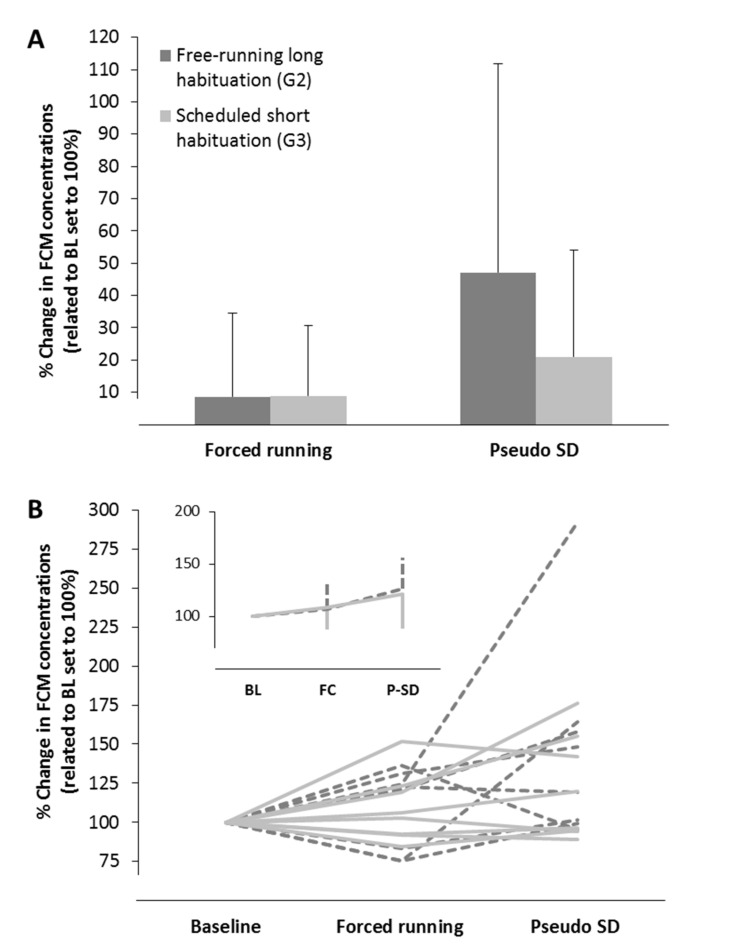
Percentage differences in concentrations of fecal corticosterone metabolites (FCMs) related to baseline (BL) values in rats with a free-running long habituation (*n* = 8) and in rats with a scheduled short habituation (*n* = 8) to activity wheels. Forced running (FC) represents housing in activity wheels during the active period (lights off) whereas pseudo-sleep deprivation (P-SD) depicts housing during the inactive period (lights on). Error bars denote standard deviations. (**A**) Mean differences in FCMs of all animals per group (*n* = 8 each). (**B**) Individual FCM courses of all animals. Dotted dark lines represent animals with long free-running habituation to the activity wheels; solid light lines depict animals with short scheduled habitation to the activity wheels. Note that one animal with the long free-running habituation showed an extreme increase in FCM levels during pseudo-SD. Insert represents mean FCM concentrations at the different timepoints in both groups after exclusion of this animal.

Effects of different habituation procedures to the activity wheels, used for SD in the test group, on FCM concentrations are shown in Figure 7. Both control groups showed no significant changes in FCM levels compared to BL (control group: 8.7 ± 26%, activity wheel control group: 8.9 ± 21.8%) during the first 12 h in the activity wheels (active phase of the animals = forced running). FCM levels were independent of the previous habituation procedure. In both control groups, a further slight increase in FCM concentrations (control group: 47.2 ± 64.7%, activity wheel control group: 21 ± 33.2%) during the second 12 h in the activity wheels (pseudo-SD during the inactive phase of the animals = motor activation without SD) could be observed. This increase was more pronounced in rats which underwent the free-running long habituation (control group G2, Figure 7A). Statistical analysis with a repeated-measures ANOVA indicated a significant effect of time (F(1.3, 18.9) = 5.5, *p* = 0.022), whereas group (F(1,14) = 0.65, *p* = 0.43) and interaction time x group (F(1.3, 18.9) = 1, *p* = 0.35) were not significant. However, detected significances between the different timepoints did not withstand post-hoc analysis. Further analysis of individual FCM courses revealed that solely one animal was responsible for elevated FCM levels during pseudo-SD in rats with the free-running long habituation. This animal showed an increase in FCMs during pseudo-SD of more than 130% in comparison to the mean of all other animals. Figure 7B depicts results after exclusion of this animal, showing only slight changes in FCM levels (26.5 ± 29.6%) due to pseudo-SD, which mirrors results in rats with the scheduled short habituation.

### 3.4. Assessment of Welfare via Score Sheets and Correlation to Fecal Corticosterone Metabolites

#### 3.4.1. Test Group G1

**Figure 8:**
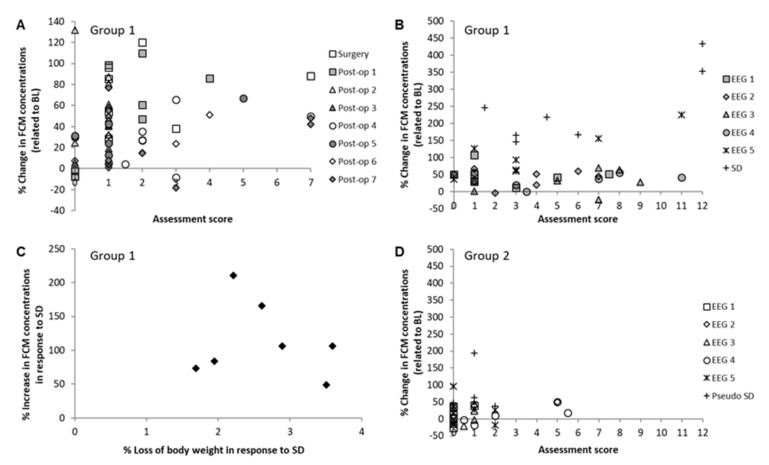
Relationship between changes in fecal corticosterone metabolite (FCM) concentrations and parameters of welfare assessment. Each category of symbols represents one day, and each symbol by itself depicts the score of one individual animal. All sleep deprivation (SD) and pseudo-SD FCM values were corrected for circadian variation. (**A**) Overall assessment scores determined on the basis of standardized score sheets in relation to changes in FCM levels during the one-week post-operative period after electroencephalograph (EEG) electrode implantation (test group G1, *n* = 7). (**B**) Overall assessment scores determined on the basis of standardized score sheets in relation to changes in FCM levels during EEG-recordings and SD in activity wheels (AW) (test group G1, *n* = 7). (**C**) Loss of body weight and increase in FCMs due to SD. Each symbol represents data of one animal. Increase in FCMs and loss of body weight were related to values determined directly before SD (test group G1, *n* = 7). (**D**) Overall assessment scores determined on the basis of standardized score sheets in relation to changes in FCMs during EEG-cage housing without EEG-recordings and pseudo-SD in AWs (control group G2, *n* = 6).

Assessment scores—mirroring the subjective impression of animals’ welfare—generally indicated low stress levels, with median scores of 1–2 (individual maximum: 7) on all investigated post-operative days (see Figure 8A). Typically, slightly abnormal behavior (less motion and reduced explorative behavior as well as more cautious movements of the head) occurred during the first days after surgery. Impaired wound healing with slight signs of inflammation in the form of redness was observed in some animals around one week after surgery. During EEG-cage housing, visual assessment scores were higher (median scores of 1–7), with maximum scores of 11 on Days 4 and 5 due to impaired wound healing along with an affected general condition. Median visual assessment score for the SD condition was 4.5, with individual maximum scores of 12 in two animals (see Figure 8B). No animal included in the study reached endpoint criteria or was found dead.

Body weight of the animals during post-operative care was constant (Figure 5B). Subsequent EEG-recordings did not result in loss of body weight, in contrast to SD in activity wheels, which significantly decreased the animals’ body weights in comparison to pre-SD condition (*p* < 0.01, Figure 5B).

There was no correlation between body weight and increase in FCM concentrations during all days of post-operative care in the test group G1 (see Table 1, top). As Figure 8C displays, loss of body weight and increase in FCMs due to SD did not correlate significantly (r = 0.2, *p* = 0.66) either. Furthermore, there was also no significant correlation between the visual assessment scores and the individual FCM levels on any day of observation (see Table 1, bottom).

Given are correlation coefficients and respective *p*-values for changes in fecal corticosterone metabolite (FCM) concentrations and body weight during the first week after implantation of EEG-electrodes (G1, top) and for changes in absolute fecal corticosterone metabolites and absolute assessment scores during the whole course of experiments (G1 and G2, bottom). Changes in FCM concentrations and body weight were normalized to baseline values set to 100%. G1: *n* = 7/8; G2: *n* = 6; BL, Baseline; EEG, electroencephalography; FCMs, fecal corticosterone metabolites; SD, sleep deprivation.

#### 3.4.2. Control Group G2

As Figure 8D depicts, assessment scores in the EEG-cage and activity wheel (AW) control group G2 were, in general, much lower than for the animals with EEG-recordings and SD in G1. Summed scores were between 0 and 2, resulting in slight variations in general condition, spontaneous behavior, and clinical findings for most animals and days. Solely one animal on EEG-cage Day 2 and two animals on EEG-cage Day 4 reached scores of 5 and 5.5, respectively. These scores were given due to dull coat and unusual behavior (extremely calm without any exploration). The following median scores were reached: EEG 1 = 0, EEG 2 = 0, EEG 3 = 0.75, EEG 4 = 1.5, EEG 5 = 0.5 and AW (pseudo-SD) = 1. There was no correlation between individual assessment scores and FCM concentrations (see Table 1, bottom).

## 4. Discussion

In the present study, individual stress levels of laboratory rats undergoing distinct experimental conditions, including surgery for EEG electrode implantation, EEG-recording in dedicated EEG-cages, and sleep deprivation (SD) in motorized activity wheels, were investigated. Of particular interest were post-operative stress levels of laboratory rats, their development over several days, and the relationship of objective (FCMs and body weight) and subjective (score sheets) monitoring parameters during different experimental procedures. As these different procedures required specific housing conditions, their influence on the animals’ welfare was investigated in separate groups.

This study shows that EEG-electrode implantation in rats resulted in a significant post-operative increase in FCM levels that declined slowly but not entirely towards BL concentrations within one week after surgery. During post-operative care, the highest FCM concentrations were measured in the samples representing surgery and early post-operative phase, indicating that the highest GC excretion by the adrenal glands occurred in direct relation to surgery. It is known that isoflurane anesthesia transiently affects corticosterone concentrations [39] but, due to its fast elimination and low solubility coefficients, this effect is of short duration and thus minor influence. In order to minimize post-operative pain, animals were treated with carprofen s.c. once a day [40]. Carprofen has been shown not to influence FCM levels [7]. Thus, the increase in FCMs after surgery is very likely to reflect actual stress levels. Increased values are likely attributable to post-operative pain as trepanation of the skull and insertion of screws for fixation of the EEG-electrodes are principally painful interventions [12] as the periosteum and the meninges are very sensitive to pain. Although, in soft tissue surgery, carprofen and the opioid tramadol, either alone or in combination, proved to be an effective pain management [41], Ciuffreda et al. [42] showed that a multi-modal treatment with carprofen (30 min pre-operative) and tramadol (one and two hours post-operatively) was more effective in a highly traumatic procedure (open chest myocardial injury). A combination of carprofen and an opioid might therefore improve analgesia after trepanation of the skull. Clearly, further studies are needed to refine pain management, particularly in preclinical studies that include surgery.

After 8 days of post-operative care, animals were placed in dedicated EEG-cages and tethered EEG-recording was started. Although FCM levels increased significantly, related to BL due to EEG-recording procedures, there was no correlation of FCMs between EEG Day 1 and late post-operative (Day 6/7) values (increase of 13.4 ± 38.1%). Thus, higher FCMs in the test group during the first day of EEG-recording might, at least partly, be attributable to generally elevated FCM levels in post-surgery animals. In addition, in control animals, new housing conditions themselves caused small but stable changes in FCMs (12.6 ± 24.5% at Day 1) in the same range as in test animals between the late post-operative period and the start of EEG-recording. In the course of EEG-recording (Day 2–4), FCM concentrations in test animals declined and leveled around late post-operative values, indicating that tethered EEG-recording might only exert minor additional stress on the animals. This observation is underpinned by a comparison between tethered and telemetric monitoring in a rat model of electrical post-status epilepticus showing only a minor impact of tethered EEG-recording [43].

At Day 5 of EEG-recording, animals were transported to the imaging facility, where a PET measurement under isoflurane anesthesia was performed. This procedure led to a noticeable and unexpected increase in FCMs in the same range as the increase due to surgery. Transportation of the animals from the housing facility to the imaging laboratories might have contributed to elevated FCM concentrations [44,45]. However, interactions between isoflurane and the HPA axis are more likely responsible for this striking increase as PET procedures (induction of isoflurane anesthesia and puncture of a tail vein under deep anesthesia) can be classified as mild [46].

Forced running in activity wheels under continuous tethered EEG-recording caused a significant elevation in FCM levels, especially during the inactive phase along with SD. Exercise control groups showed that both new housing conditions and motor activation by forced running had only a low impact on FCMs. Interestingly, extensive habituation procedures to the used activity wheels did not have any effect.

Sleep loss by itself is a physiological stressor in humans [47]. In rats, SD can be performed by various methods, all of which involve exposing animals to an additional stress (for review, see Nollet et al. [48]). Most popular methods, such as gentle handling, the use of treadmills and platforms, as well as slowly rotating activity wheels, caused a significant activation of the HPA axis in the majority of previous studies [49,50,51,52,53], thus supporting our results. To the best of our knowledge, so far, no study has investigated SD in combination with tethered EEG-recording—a method frequently used to quantify SD. Meerlo et al. [54] detected increases in plasma corticosterone concentrations due to acute SD (activity wheels; no EEG-recording) of approximately 300% above BL values. This increase is in the same range which we observed in the current study. It further indicates that EEG-recording during SD does not pose significant additional effects. We therefore assume that activity-wheel-induced SD itself is primarily responsible for the significant increase in FCM concentrations. SD effects in the brain seem to be mediated by adenosine, as Kalinchuk et al. [55] demonstrated by significantly elevated adenosine levels in rats’ basal forebrain and frontal cortex. Adenosine in turn is able to stimulate corticosterone secretion [56].

However, with regard to refinement, two recently published studies showed no significant elevations of plasma GC after SD with the use of a rotating drum [57] and a specialized air puff feedback system [58]. Notably, the latter was ineffective in producing SD of more than 5 h and should be adapted when used for longer SD protocols. Genetic sleep deprivation techniques such as chemogenetic activation/inactivation of sleep-related neuronal circuits are another, though technically challenging, option. These techniques are potentially prone to undesired epiphenomena or misleading results by interference with brain regions and circuits that are not involved in sleep–wake regulation or the HPA axis (for review, see Nollet et al. [48]).

Measuring changes in body weight is commonly used for the assessment of post-operative recovery in laboratory rodents [59,60]. In the present study, rats showed only minimal losses of body weight during post-operative recovery, while FCM values clearly indicated the presence of elevated stress levels. These findings are corroborated by studies in a mouse model of depression, where animals were treated with short-lasting food shocks, showing transient elevations of corticosterone but no weight losses [23]. The mismatch between FCMs and body weight might be caused by the relatively short effect duration of the stressor, since chronically repeated restrained stress results in chronically elevated serum corticosterone concentrations and long-lasting decreases in body weight both in mice [61] and rats [62]. Thereby, chronic stress and thus elevated GC levels might promote more intense physiological changes, probably by reducing food intake via modification of canonical food-intake-related genes [61]. A number of experimental conditions with short-term stress effects are therefore probably not adequately monitored by pure visual inspection and phenomenological assessment.

The observed increase in FCM concentrations after SD was in line with a significant loss of body weight. However, there was no correlation between individual loss of body weight and individual increase in FCM levels. As the sampling period for SD was restricted to 12 h, FCM data might be slightly biased as data had to be corrected for circadian variations in FCM excretion. Nevertheless, SD massively increased FCM concentration (247% in comparison to BL values) while, in contrast, the circadian rhythmicity most likely plays a minor role, with a detected increase of 18%. Unfortunately, it was not possible to extend the sampling interval after SD because of the bias induced by anesthesia of repeated PET measurements. Therefore, the recovery period after SD was not investigated.

Behavioral assessment based on score sheets showed low scores in the present study when compared with elevated FCM values. On the one hand, elevated FCM levels might reflect a successful stress response, resulting in low visual scores during welfare assessment. On the other hand, although assessment of animals by means of standardized score sheets and by various persons is common practice in animal laboratories, it is, however, questionable whether this procedure accurately reflects the welfare of animals under all given conditions. Such evaluations are obviously very explorative since they are subjective and less standardized due to interrater variability. Moreover, evaluation by means of the score sheets reflects only one single point of time, whereas 24-h sampling periods of FCMs comprise several hours and might therefore be more sensitive and adequate for monitoring of longer time periods. In the present study, low post-surgical levels of stress might not have created visible changes in behavior during the specific time points when the experimenter was in the room, and especially as observation took place during the inactive, lights-on phase of the animals. A comparable phenomenon was already observed by Adamson et al. [7]. In general, video recordings combined with remote scoring might allow for a more reliable assessment of animals’ welfare, especially when scoring is performed during the active period of the animals. An accordingly designed automatic camera-based monitoring system has recently been established in anesthetized animals but not yet been validated for awake animals [63]. Further, as proposed by Jacobson et al. [9] and shown by Pfeiffenberger et al. [12], scoring of specific, pain-related behavioral events in combination with monitoring of food and water intake is likely to be a more sensitive parameter for the detection of pain and stress after surgery. This is also true for the mouse [64] and rat [65] grimace scales, although the evaluation of facial expressions demands a well-adjusted camera system and might be challenging when animals are housed in groups. Another option for a more sophisticated welfare assessment, additionally coming along with a certain refinement, might be an observation of voluntary wheel running (VWR) behavior, as recently suggested and investigated in a mouse model of colitis [22] and learned helplessness induced by electric foot shocks [23]. However, as motor activation has often an overall effect on the progression of disease or recovery (e.g., in the chronic unpredictable mild stress model of depression [66]), VWR might not be applicable in general.

In order to determine individual stress levels of certain time periods, all feces voided during each sampling interval were collected. This measure prevents misleading results because the FCM content is highly variable in individual fecal pellets [37,67]. Additionally, sampling intervals were set to 24 h for most of the experiments in order to eliminate the effects of circadian variations in GC excretion [28,38]. The delay in fecal peak FCM excretion in rats is approximately 14.8 ± 2.4 h [28]. Surgeries as well as experimental interventions were timed to take place at the beginning of each 24-h sampling interval, ensuring that peak FCM excretion occurred in the same sampling interval as the event to be investigated. Thus, a reliable assessment of the overall stress levels of the animals was ensured.

Although all efforts were made to habituate animals to experimental conditions (handling, feces sampling) and to keep environmental conditions constant (temperature, light–dark cycle, etc.), basal FCM levels considerably varied between animals at coefficients of variance of approximately 30% for all groups. In order to minimize the influence of inter-individual variations, only intra-individual differences in FCMs which were related to respective BL values were applied for statistical comparisons. BL FCM concentrations were defined as average of two consecutive days to minimize the risk of unreliable baseline values. Statistical comparisons of baseline FCM concentrations of both days showed that BL values were stable and reliable within each animal. Furthermore, analyses of BL FCM concentrations between groups revealed no statistical differences, which supports the view of highly reliable reference FCM levels.

## 5. Conclusions

Our results showed that, under standard experimental conditions with relatively subtle variations in the animals’ welfare status, score sheets for visual assessment were less reliable in reflecting physiological stress responses compared to FCM measurements. Changes in body weight as a more objective parameter were small and did not mirror relatively slight stress responses as seen in the present study. As visual assessment is a method allowing immediate interventions on suffering animals to alleviate burden, it is essential that scoring is carried out carefully and with controlled rules, e.g., thorough training, by including inter observer reliability assessment or entrusting one single expert, ideally blinded to experimental conditions.

Moreover, the current standard of timely stress assessment in experimental rodents should be ideally complemented by validated objective measures (e.g., fecal FCM measured by well-established assays for reliable detection of FCMs) to improve the tracking of stress levels for a comprehensive appraisal of the animals’ welfare status in a retrospective manner and to refine stressor procedures in the long run.

## Figures and Tables

**Table 1:** Correlation coefficients for changes in fecal corticosterone metabolites (FCMs), body weight, and visual assessment scores.

Changes in FCM Concentrations and Body Weight during Post-Operative Care (Pearson Correlation Coefficients and (*p*-Values))		
Day	G1 (test group)	
Post-op 1	−0.02 (0.97)	
Post-op 2	−0.79 (0.06)	
Post-op 3	−0.11 (0.81)	
Post-op 4	−0.39 (0.39)	
Post-op 5	0.22 (0.63)	
Post-op 6	0.54 (0.21)	
Post-op 7	0.13 (0.77)	
**Absolute FCM Concentrations and Assessment Scores during Different Interventions (Kendall’s Tau Rank Correlation** **Coefficient and (*p*-Values)**		
Day	G1 (test group)	G2 (control group)
Surgery	−0.36 (0.25)	
Post-op 1	0.08 (0.79)	
Post-op 2	0.11 (0.74)	
Post-op 3	−0.07 (0.83)	
Post-op 4	−0.23 (0.44)	
Post-op 5	0.04 (0.89)	
Post-op 6	0.24 (0.43)	
Post-op 7	0.37 (0.23)	
EEG 1	0.42 (0.16)	−0.24 (0.66)
EEG 2	−0.04 (0.90)	−0.26 (0.50)
EEG 3	0.27 (0.38)	0.23 (0.54)
EEG 4	−0.40 (0.17)	0.07 (0.85)
EEG 5 (PET in G1)	0.32 (0.29)	−0.55 (0.15)
SD (G1)/Pseudo-SD (G2)	0.55 (0.09)	−0.08 (0.84)

## Data Availability

The data presented in this study are available in supplementary material Data S1.

## Supplementary Materials

The following are available online at https://www.mdpi.com/2076-2615/11/3/710/s1, File S1: Calculation of circadian bias for a 12-h sampling period with sampling time at midnight to detect effects of 12-h sleep deprivation, Figure S1: Circadian rhythmicity of fecal corticosterone metabolite (FCM) concentrations analyzed one and two weeks after end of shifting of the circadian cycle to lights-off from noon until midnight, Table S1: Absolute fecal corticosterone metabolite (FCM) concentrations and calculation of bias for a 12-h sampling period with sampling time at midnight, Data S1: Underlying raw data of presented results.

## References

[1] Russel W., Burch R. (1959). The Principles of Humane Experimental Technique.

[2] (2010). Directive 2010/63/EU of the European Parliament and of the Council of 22 September 2010 on the protection of animals used for scientific purposes. Off. J. Eur. Union..

[3] Van Vlissingen J.F., Borrens M., Girod A., Lelovas P., Morrison F., Torres Y.S. (2015). The reporting of clinical signs in laboratory animals: FELASA Working Group Report. Lab. Anim..

[4] Morton D.B., Griffiths P.H. (1985). Guidelines on the recognition of pain, distress and discomfort in experimental animals and an hypothesis for assessment. Vet. Rec..

[5] (2012). National Competent Authorities for the implementation of Directive 2010/63/EU on the protection of animals used for scientific purposes—Working document on a severity assessment framework. Eur. Comm..

[6] Fraser D., Ritchie J.S., Fraser A.F. (1975). The term “stress” in a veterinary context. Br. Vet. J..

[7] Adamson T.W., Kendall L.V., Goss S., Grayson K., Touma C., Palme R., Chen J.Q., Borowsky A.D. (2010). Assessment of carprofen and buprenorphine on recovery of mice after surgical removal of the mammary fat pad. J. Am. Assoc. Lab. Anim. Sci..

[8] Goldschlager G.B., Gillespie V.L., Palme R., Baxter M.G. (2013). Effects of multimodal analgesia with low-dose buprenorphine and meloxicam on fecal glucocorticoid metabolites after surgery in New Zealand white rabbits (Oryctolagus cuniculus). J. Am. Assoc. Lab. Anim. Sci..

[9] Jacobsen K.R., Kalliokoski O., Teilmann A.C., Hau J., Abelson K.S.P. (2012). Postsurgical food and water consumption, fecal corti-costerone metabolites, and behaviour assessment as noninvasive measures of pain in vasectomized BALB/c mice. J. Am. Assoc. Lab. Anim. Sci..

[10] Kalliokoski O., Jacobsen K.R., Darusman H.S., Henriksen T., Weimann A., Poulsen H.E., Hau J., Abelson K.S.P. (2013). mice do not habituate to metabolism cage housing–a three week study of male BALB/c mice. PLoS ONE.

[11] Palme R. (2019). Non-invasive measurement of glucocorticoids: Advances and problems. Physiol. Behav..

[12] Pfeiffenberger U., Yau T., Fink D., Tichy A., Palme R., Egerbacher M., Rülicke T. (2015). Assessment and refinement of intra-bone marrow transplantation in mice. Lab. Anim..

[13] Royo F., Björk N., Carlsson H.E., Mayo S., Hau J. (2004). Impact of chronic catheterization and automated blood sampling (Ac-cusampler) on serum corticosterone and fecal immunoreactive corticosterone metabolites and immunoglobulin A in male rats. J. Endocrinol..

[14] Sundbom R., Jacobsen K.R., Kalliokoski O., Hau J., Abelson K.S.P. (2011). Post-operative corticosterone levels in plasma and feces of mice subjected to permanent catheterization and automated blood sampling. In Vivo.

[15] Zieglowski L., Kümmecke A., Ernst L., Schulz M., Talbot S.R., Palme R., Czaplik M., Tolba R.H. (2020). Severity assessment using three common behavioral or locomotor tests after laparotomy in rats: A pilot study. Lab. Anim..

[16] Keubler L.M., Hoppe N., Potschka H., Talbot S.R., Vollmar B., Zechner D., Häger C., Bleich A. (2020). Where are we heading? Challenges in evidence-based severity assessment. Lab. Anim..

[17] Bodden C., Siestrup S., Palme R., Kaiser S., Sachser N., Richter S.H. (2018). Evidence-based severity assessment: Impact of repeated versus single open-field testing on welfare in C57BL/6J mice. Behav. Brain Res..

[18] Hohlbaum K., Bert B., Dietze S., Palme R., Fink H., Thöne-Reineke C. (2017). Severity classification of repeated isoflurane anesthesia in C57BL/6JRj mice—Assessing the degree of distress. PLoS ONE.

[19] Kumstel S., Wendt E.H.U., Eichberg J., Talbot S.R., Häger C., Zhang X., Abdelrahman A., Schönrogge M., Palme R., Bleich A. (2020). Grading animal distress and side effects of therapies. Ann. N. Y. Acad. Sci..

[20] Meyer N., Kröger M., Thümmler J., Tietze L., Palme R., Touma C. (2020). Impact of three commonly used blood sampling techniques on the welfare of laboratory mice: Taking the animal’s perspective. PLoS ONE.

[21] Wright-Williams S.L., Courade J.-P., Richardson C.A., Roughan J.V., Flecknell P.A. (2007). Effects of vasectomy surgery and meloxicam treatment on faecal corticosterone levels and behaviour in two strains of laboratory mouse. Pain.

[22] Häger C., Keubler L.M., Talbot S.R., Biernot S., Weegh N., Buchheister S., Buettner M., Glage S., Bleich A. (2018). Running in the wheel: Defining individual severity levels in mice. PLoS Biol..

[23] Mallien A.S., Häger C., Palme R., Talbot S.R., Vogt M.A., Pfeiffer N., Brandwein C., Struve B., Inta D., Chourbaji S. (2020). Systematic analysis of severity in a widely used cognitive depression model for mice. Lab. Anim..

[24] Nicolaides N.C., Kyratzi E., Lamprokostopoulou A., Chrousos G.P., Charmandari E. (2015). Stress, the Stress System and the Role of Glucocorticoids. Neuroimmunomodulation.

[25] Möstl E., Palme R. (2002). Hormones as indicators of stress. Domest. Anim. Endocrinol..

[26] Christiansen S., Bouzinova E.V., Palme R., Wiborg O. (2012). Circadian activity of the hypothalamic-pituitary-adrenal axis is differentially affected in the rat chronic mild stress model of depression. Stress.

[27] Palme R. (2012). Monitoring stress hormone metabolites as a useful, non-invasive tool for welfare assessment in farm animals. Anim. Welf..

[28] Lepschy M., Touma C., Hruby R., Palme R. (2007). Non-invasive measurement of adrenocortical activity in male and female rats. Lab. Anim..

[29] Touma C., Sachser N., Möstl E., Palme R. (2003). Effects of sex and time of day on metabolism and excretion of corticosterone in urine and feces of mice. Gen. Comp. Endocrinol..

[30] Touma C., Palme R., Sachser N. (2004). Analyzing corticosterone metabolites in fecal samples of mice: A noninvasive technique to monitor stress hormones. Horm. Behav..

[31] Christie M.A., McKenna J.T., Connolly N.P., McCarley R.W., Strecker R.E. (2008). 24 hours of sleep deprivation in the rat increases sleepiness and decreases vigilance: Introduction of the rat-psychomotor vigilance task. J. Sleep Res..

[32] Kroll T., Elmenhorst D., Weisshaupt A., Beer S., Bauer A. (2014). Reproducibility of non-invasive A1 adenosine receptor quantification in the Rat Brain Using [18F]CPFPX and positron emission tomography. Mol. Imaging Biol..

[33] Gong H., McGinty D., Guzman-Marin R., Chew K.T., Stewart D., Szymusiak R. (2004). Activation of c-fos in GABAergic neurons in the preoptic area during sleep and in response to sleep deprivation. J. Physiol..

[34] Guzmán-Marín R., Suntsova N., Stewart D.R., Gong H., Szymusiak R., McGinty D. (2003). Sleep deprivation reduces proliferation of cells in the dentate gyrus of the hippocampus in rats. J. Physiol..

[35] Kanzler S., Rix A., Czigany Z., Tanaka H., Fukushima K., Kögel B., Pawlowsky K., Tolba R.H. (2016). Recommendation for severity assessment following liver resection and liver transplantation in rats: Part I. Lab. Anim..

[36] Pinkernell S., Becker K., Lindauer U. (2016). Severity assessment and scoring for neurosurgical models in rodents. Lab. Anim..

[37] Palme R., Touma C., Arias N., Dominchin M.F., Lepschy M. (2013). Steroid extraction: Get the best out of faecal samples. Wien. Tierärztliche Mon..

[38] Lepschy M., Touma C., Palme R. (2010). Faecal glucocorticoid metabolites: How to express yourself—Comparison of absolute amounts versus concentrations in samples from a study in laboratory rats. Lab. Anim..

[39] Altholtz L.Y., Fowler K.A., Badura L.L., Kovacs M.S. (2006). Comparison of the stress response in rats to repeated isoflurane or CO2:O2 anesthesia used for restraint during serial blood collection via the jugular vein. J. Am. Assoc. Lab. Anim. Sci..

[40] Henke J., Haberstroh J., Sager M., Becker K., Eberspächer E., Bergadano A., Zahner D., Arras M. (2015). Pain Management for Laboratory Animals—Expert Information: Committee on Anaesthesia of GV-SOLAS.

[41] Zegre Cannon C., Kissling G.E., Goulding D.R., King-Herbert A.P., Blankenship-Paris T. (2011). Analgesic effects of tramadol, carprofen or multimodal analgesia in rats undergoing ventral laparotomy. Lab. Anim..

[42] Ciuffreda M.C., Tolva V., Casana R., Gnecchi M., Vanoli E., Spazzolini C., Roughan J., Calvillo L. (2014). Rat Experimental model of myocardial ischemia/reperfusion injury: An ethical approach to set up the analgesic management of acute post-surgical pain. PLoS ONE.

[43] Seiffert I., Van Dijk R.M., Koska I., Di Liberto V., Möller C., Palme R., Hellweg R., Potschka H. (2019). Toward evidence-based severity assessment in rat models with repeated seizures: III. Electrical post-status epilepticus model. Epilepsia.

[44] Armario A., Montero J.L., Balasch J. (1985). Sensitivity of corticosterone and some metabolic variables to graded levels of low in-tensity stresses in adult male rats. Physiol. Bahav..

[45] Tuli J.S., Smith J.A., Morton D.B. (1995). Stress measurements in mice after transportation. Lab. Anim..

[46] Rix A., Drude N., Mrugalla A., Mottaghy F.M., Tolba R.H., Kiessling F. (2019). Performance of severity parameters to detect chemotherapy-induced pain and distress in mice. Lab. Anim..

[47] Spiegel K., Leproult R., Van Couter E. (1999). Impact of sleep debt on metabolic and endocrine function. Lancet.

[48] Nollet M., Wisden W., Franks N.P. (2020). Sleep deprivation and stress: A reciprocal relationship. Interface Focus.

[49] Coenen A.M., Van Luijtelaar E.L. (1985). Stress induced by three procedures of deprivation of paradoxical sleep. Physiol. Behav..

[50] Hairston I.S., Ruby N.F., Brooke S., Peyron C., Denning D.P., Heller H., Sapolsky R.M. (2001). Sleep deprivation elevates plasma corticosterone levels in neonatal rats. Neurosci. Lett..

[51] Penalva R.G., Lancel M., Flachskamm C., Reul J.M., Holsboer F., Linthorst A.C. (2003). Effect of sleep and sleep deprivation on serotonergic neurotransmission in the hippocampus: A combined in vivo microdialysis/EEG study in rats. Eur. J. Neurosci..

[52] Sgoifo A., Buwalda B., Roos M., Costoli T., Merati G., Meerlo P. (2006). Effects of sleep deprivation on cardiac autonomic and pituitary-adrenocortical stress reactivity in rats. Psychoneuroendocrinology.

[53] Tartar J.L., Ward C.P., Cordeira J.W., Legare S.L., Blanchette A.J., McCarley R.W., Strecker R.E. (2009). Experimental sleep fragmentation and sleep deprivation in rats increases exploration in an open field test of anxiety while increasing plasma corticosterone levels. Behav. Brain Res..

[54] Meerlo P., Koehl M., Van Der Borght K., Turek F.W. (2002). Sleep restriction alters the hypothalamic-pituitary-adrenal response to stress. J. Neuroendocr..

[55] Kalinchuk A.V., McCarley R.W., Porkka-Heiskanen T., Basheer R. (2011). The time course of adenosine, nitric oxide (NO) and in-ducible NO synthase changes in the brain with sleep loss and their role in the non-rapid eye movement sleep homeostatic cascade. J. Neurochem..

[56] Scaccianoce S., Navarra D., Di Sciullo A., Angelucci L., Endröczi E. (1989). Adenosine and pituitary-adrenocortical axis activity in the rat. Neuroendocrinology.

[57] Leenaars C.H., Dematteis M., Joosten R.N., Eggels L., Sandberg H., Schirris M., Feenstra M.G., Van Someren E.J. (2011). A new automated method for rat sleep deprivation with minimal confounding effects on corticosterone and locomotor activity. J. Neurosci. Methods.

[58] Gross B.A., Vanderheyden W.M., Urpa L.M., Davis D.E., Fitzpatrick C.J., Prabhu K., Poe G.R. (2015). Stress-free automatic sleep deprivation using air puffs. J. Neurosci. Methods.

[59] Martini L., Lorenzini R.N., Cinotti S., Fini M., Giavaresi G., Giardino R. (2000). Evaluation of pain and stress levels of animals used in experimental research. J. Surg. Res..

[60] Shavit Y., Fish G., Wolf G., Mayburd E., Meerson Y., Yirmiya R., Beilin B. (2005). The effects of perioperative pain management techniques on food consumption and body weight after laparotomy in rats. Anesthesia Analg..

[61] Jeong J.Y., Lee D.H., Kang S.S. (2013). Effects of chronic restraint stress on body weight, food intake, and hypothalamic gene expressions in mice. Endocrinol. Metab..

[62] Harris R.B.S., Zhou J., Youngblood B.D., Rybkin I.I., Smagin G.N., Ryan D.H. (1998). Effect of repeated stress on body weight and body composition of rats fed low- and high-fat diets. Am. J. Physiol..

[63] Kunczik J., Pereira C.B., Zieglowski L., Tolba R., Wassermann L., Häger C., Bleich A., Janssen H., Thum T., Czaplik M. (2019). Remote vitals monitoring in rodents using video recordings. Biomed. Opt. Express.

[64] Leach M.C., Klaus K., Miller A.L., Di Perrotolo M.S., Sotocinal S.G., Flecknell P.A. (2012). The Assessment of Post-Vasectomy Pain in Mice Using Behaviour and the Mouse Grimace Scale. PLoS ONE.

[65] Sotocinal S.G., Sorge R.E., Zaloum A., Tuttle A.H., Martin L.J., Wieskopf J.S., Mapplebeck J.C.S., Wei P., Zhan S., Zhang S. (2011). The rat grimace scale: A partially automated method for quantifying pain in the laboratory rat via facial expressions. Mol. Pain.

[66] Huang P., Dong Z., Huang W., Zhou C., Zhong W., Hu P., Wen G., Sun X., Hua H., Cao H. (2017). Voluntary wheel running ameliorates depression-like behaviors and brain blood oxygen level-dependent signals in chronic unpredictable mild stress mice. Behav. Brain Res..

[67] Millspaugh J.J., Washburn B.E. (2003). Within-sample variation of fecal glucocorticoid measurements. Gen. Comp. Endocrinol..

